# CT scan for suspected acute appendicitis

**DOI:** 10.3402/jchimp.v1i4.10926

**Published:** 2012-01-26

**Authors:** David M. Widlus

**Affiliations:** Union Memorial Hospital, Department of Radiology, University of Maryland School of Medicine, Baltimore, MD, USA

Appendicitis is common with a 7% lifetime risk for an individual in the United States. Mean age at diagnosis is 22 years old. While frequently clinically obvious, by 2006, more than 90% of patients diagnosed with appendicitis had a CT scan of the abdomen and pelvis performed. Use of CT scans has allowed a decrease in false-negative rate at appendectomy to under 10% from a rate of approximately 20% before routine use of CT scan. In addition, the rate of perforation has decreased from nearly 30% to under 15%. In the pediatric population, initial ultrasound is often recommended, with CT utilized if the sonogram is inconclusive (Fig. 3).

Findings at CT scan, which are suggestive or diagnostic of appendicitis, include: dilation of the appendix to more than 6 mm; thickening of the wall of the appendix; enhancement of the wall of the appendix, which can be homogeneous or heterogeneous, including the stratified appearance referred to as a target sign; peri-appendiceal inflammatory stranding; appendicolith; peri-appendiceal abscess (Figs. 1 and 2). A focal area with decreased enhancement has been shown to be a reliable sign of perforation. Sensitivity and specificity of diagnosis with CT scans are up to 98% for each. When appendicitis is not present, an alternative diagnosis can be suggested in up to 40% of cases.

**Fig. 1:**
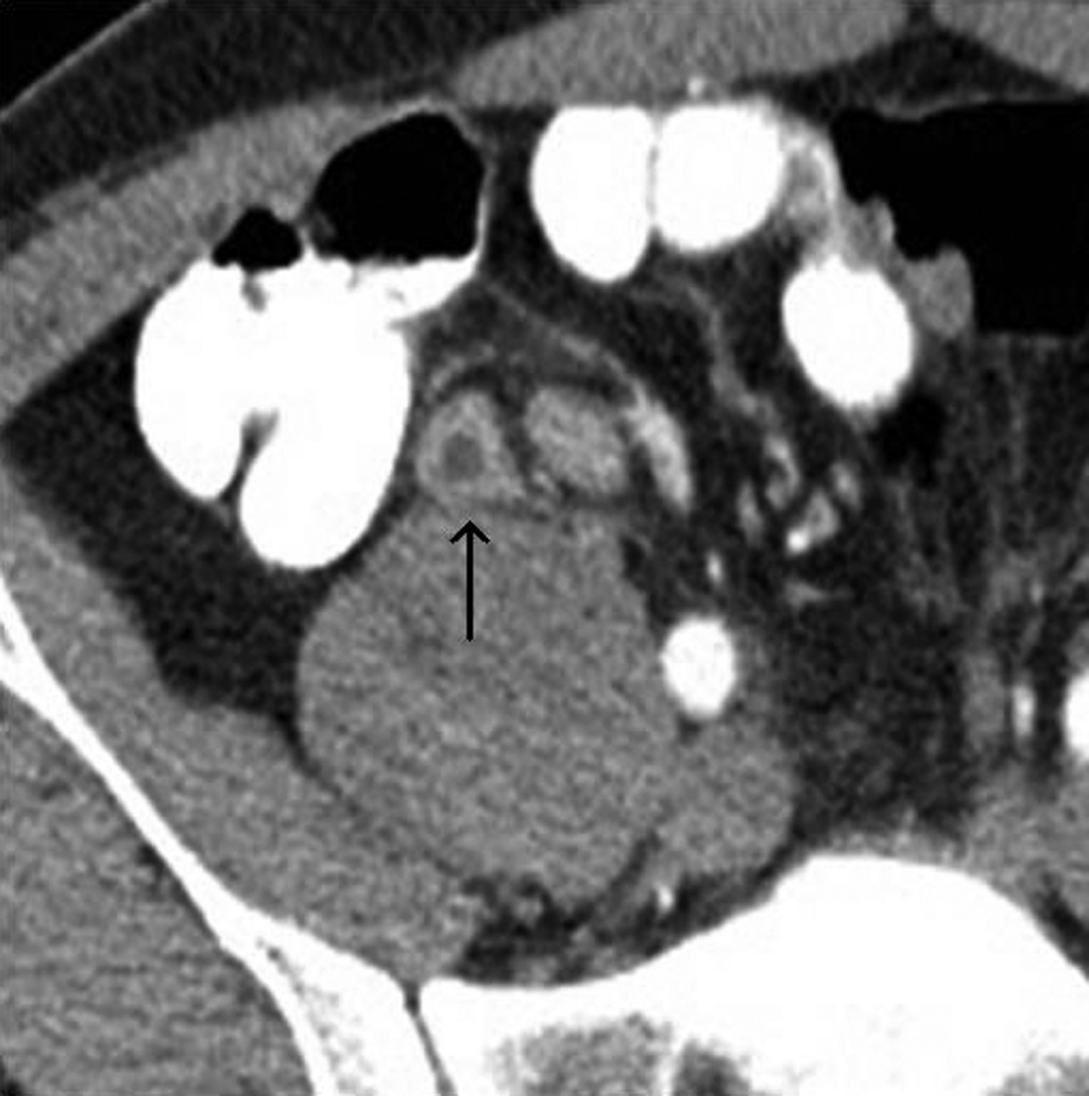
Axial CT scan shows an inflamed, thick-walled appendix with peri-appendiceal inflammatory stranding (arrow).

**Fig. 2:**
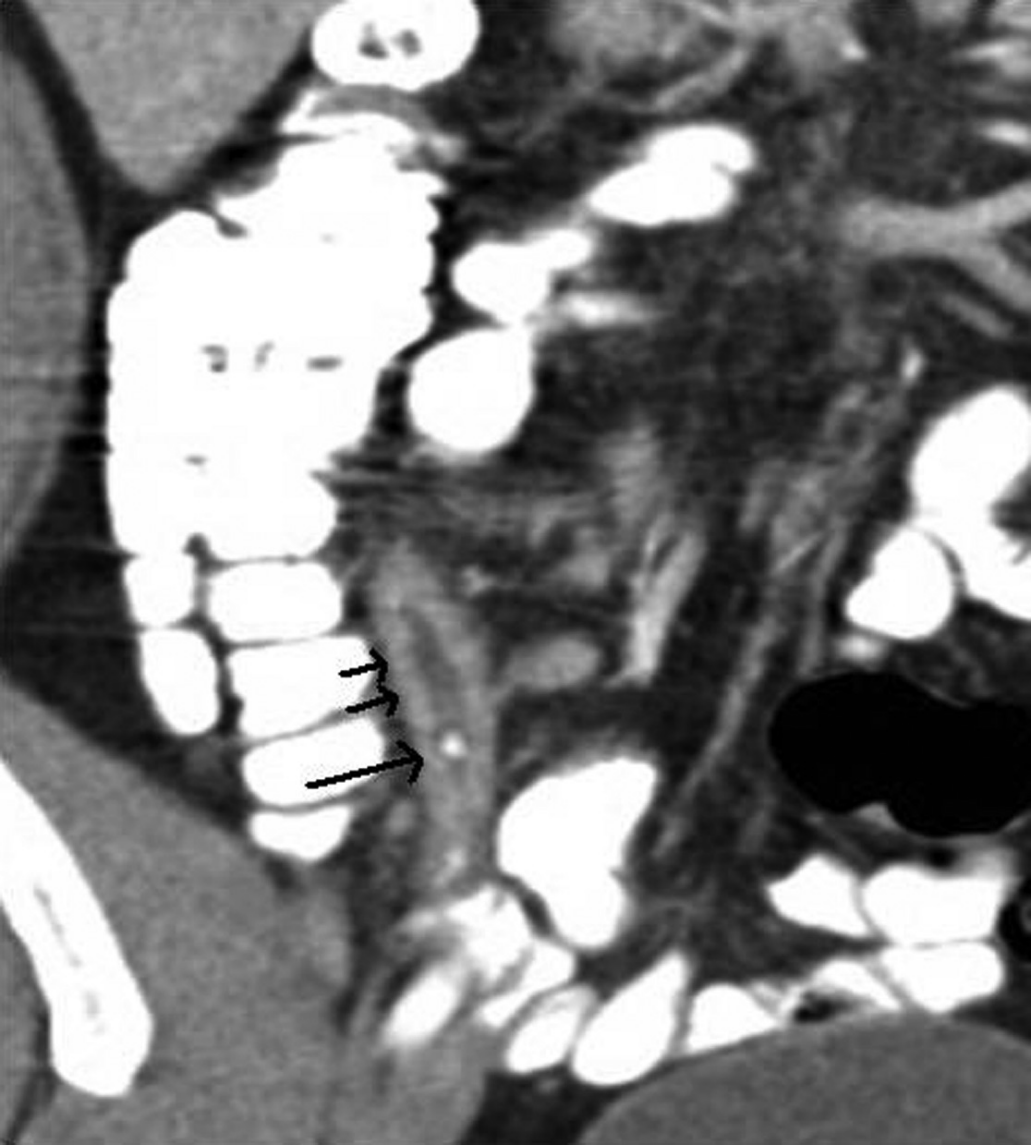
Coronal view shows the thick-walled appendix with stranding (short arrows). An appendicolith is clearly seen (long arrow).

**Fig. 3:**
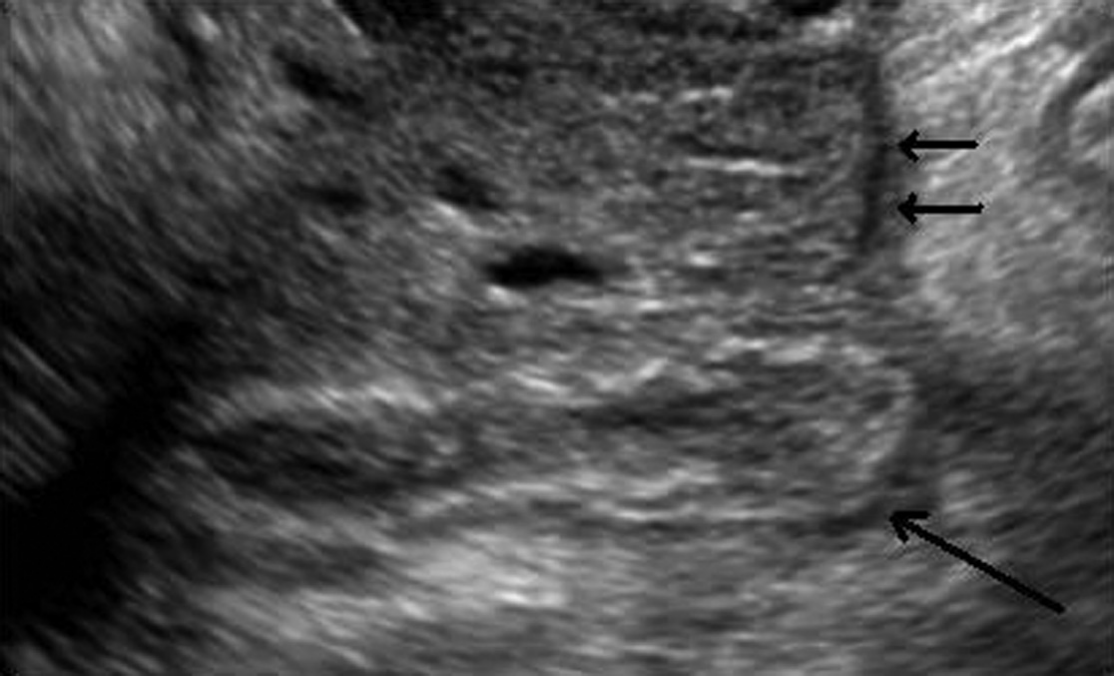
Transvaginal sonogram in a 16-year-old patient shows a thick-walled appendix with lumen distended with fluid (long arrow). A normal right ovary is seen just anterior (short arrows).

## References

[1] Pickhardt PJ, Lawrence EM, Pooler BD, Bruce RJ (2011). Diagnostic performance of multidetector computed tomography for suspected acute appendicitis. Ann Intern Med.

